# Cumulative weight reduction across repeated acupoint catgut embedding sessions in obese women: a retrospective cohort study

**DOI:** 10.3389/fmed.2026.1945172

**Published:** 2026-10-16

**Authors:** Xiaolan Ji, Xiyan Xin, Dong Li, Lili Hou, Yang Ye

**Affiliations:** Department of Traditional Chinese Medicine, Peking University Third Hospital, Beijing, China

**Keywords:** acupoint catgut embedding, body mass index, obesity, retrospective cohort study, women

## Abstract

**Introduction:**

Acupoint catgut embedding (ACE), a minimally invasive acupuncture-derived technique, is gaining popularity in weight management, yet high-quality evidence focusing specifically on obese individuals remains sparse. This retrospective cohort study was designed to assess whether ACE is associated with lower body mass index (BMI) in obese women and whether this association accumulates across repeated sessions.

**Methods:**

We included obese women (BMI ≥ 28 kg/m^2^, aged 18–60 years) who completed four ACE treatments and attended five study visits (baseline and four post-treatment assessments) at Peking University Third Hospital from January 2016 to September 2025. An untreated control group of volunteers with similar baseline age and BMI was enrolled prospectively; their weight was recorded at five biweekly visits without any intervention. Baseline variables and serial BMI measurements were compared between groups using non-parametric tests.

**Results:**

Ninety-eight participants in the ACE group and 33 participants in the control group were analysed. The two groups were similar at baseline in age, height, weight, BMI, and waist circumference. By the third, fourth, and fifth visits, the ACE group showed significantly lower BMI than the control group (*P* = 0.043, 0.001, and <0.001, respectively). Within the ACE group, BMI at visits 2 through 5 was significantly lower than baseline, suggesting a cumulative association across successive sessions.

**Conclusion:**

Some evidence from this study suggests that ACE may be associated with weight loss in obese women, with differences becoming detectable after two sessions and appearing to strengthen with additional treatments. No major adverse events were identified during the observed treatment period, supporting ACE as a possible adjunctive option for obesity care.

## 1 Introduction

Obesity has become a worldwide health crisis of staggering proportions. According to the World Obesity Federation, in 2025 about 41% of Chinese adults were classified as overweight and 9% as obese under international criteria (BMI ≥25 kg/m^2^ for overweight and BMI ≥30 kg/m^2^ for obesity). Projections suggest that by 2030, the total number of adults living with overweight or obesity in China will reach 515 million. Should China's own national diagnostic thresholds (BMI ≥24 kg/m^2^ for overweight and ≥28 kg/m^2^ for obesity) be applied, these figures would be considerably higher. Such a growing burden places immense strain on both the healthcare system and society at large (1).

The health toll of obesity is well documented. It is a major driver of hypertension, dyslipidaemia, coronary disease, stroke, and various malignancies—breast, colorectal, and endometrial cancers in particular (2). Obesity also predisposes to metabolic dysfunction-associated steatotic liver disease, reproductive disorders, and osteoarthritis (3). In women of childbearing age, it increases the risk of infertility, gestational diabetes, pre-eclampsia, preterm delivery, and adverse neonatal outcomes (4). Economically, the direct and indirect costs of obesity are staggering, further underscoring the urgency for effective interventions.

Current weight-loss strategies include lifestyle changes, pharmacotherapy, and bariatric surgery. Lifestyle modification is the bedrock, but long-term adherence is notoriously poor (5). Drugs like glucagon-like peptide-1 (GLP-1) receptor agonists can produce impressive short-term results, yet weight rebound after discontinuation is common, and access is limited by cost and side effects (6). Surgery is reserved for those with severe obesity or related comorbidities, leaving a large segment of the obese population without satisfactory options (7). This gap calls for safe, affordable, and sustainable therapies that can be widely applied.

Acupoint catgut embedding (ACE) is an innovative technique that combines traditional Chinese medicine acupoint theory with a sustained-release principle (8). By placing absorbable sutures under the skin at specific points, ACE delivers continuous low-grade stimulation over days to weeks, mimicking a prolonged acupuncture effect (9). Mechanistic studies indicate that ACE may regulate appetite-related hypothalamic nuclei, improve leptin and insulin sensitivity, modulate lipid and glucose metabolism, reshape gut microbiota, and fine-tune immune responses (10–13). Several meta-analyses have confirmed its efficacy in reducing BMI, with a recent synthesis of 23 RCTs showing a weighted mean difference of −1.72 kg/m^2^ in obese women (11). However, most trials have focused on relatively short courses or used sham controls that might not capture the full clinical effect in routine practice. Moreover, the extent to which the effect accumulates across repeated sessions—and how many sessions are needed to achieve meaningful loss—remains poorly characterised.

To fill this knowledge gap, we conducted a retrospective cohort study to evaluate the real-world effectiveness of ACE for weight reduction in obese women. Our primary aim was to quantify BMI changes after four ACE sessions, and our secondary aim was to explore whether the association with weight reduction accumulates across successive treatments.

## 2 Methods

### 2.1 Study design and setting

This was a single-centre retrospective cohort study carried out in the Traditional Chinese Medicine Outpatient Clinic of the Aesthetic Center, Peking University Third Hospital. We reviewed medical records of women who underwent ACE between January 2016 and September 2025 for the ACE group, and we prospectively enrolled an untreated comparison group of volunteers between October 2025 and May 2026. Participants in the control group met the same age and BMI eligibility criteria and were selected to have age and BMI distributions similar to those of the ACE group. The ethics committee of Peking University Third Hospital approved the protocol (M20250733).

### 2.2 Participants

Inclusion criteria for the ACE group were women aged 18–60 years with a baseline BMI ≥ 28.0 kg/m^2^ who had completed four ACE treatment sessions and attended five study visits (visit 1, baseline; visits 2–5, after each of the four ACE sessions), with no more than two months between consecutive visits, and for whom complete age and BMI data were available (14). Inclusion criteria for the control group were untreated female volunteers aged 18–60 years with a BMI ≥ 28.0 kg/m^2^, no history of concurrent weight-loss interventions, and agreement to have their weight measured every two weeks for five time points. Exclusion criteria for both groups included secondary obesity due to endocrine disorders; severe cardiovascular, hepatic, or renal disease; pregnancy or lactation; local skin infection at acupoint sites; or coagulation defects.

### 2.3 Interventions

#### 2.3.1 ACE group

All procedures were performed by one of three experienced practitioners, each with at least 5 years of practice in acupuncture and ACE. A standardized point prescription was used: RN12 (Zhongwan), RN9 (Shuifen), bilateral ST25 (Tianshu), RN6 (Qihai), RN4 (Guanyuan), bilateral ST36 (Zusanli), and bilateral BL23 (Shenshu), with up to three additional points allowed based on individual presentation. In this study, PDO (polydioxanone) sutures were used as the embedding material, the term “catgut” in the therapy name is retained as a conventional designation and does not reflect the actual suture composition. Under aseptic conditions, a 1.5-cm PDO suture was loaded into a sterile embedding needle (0.8 × 60 mm) and inserted subcutaneously to achieve deqi sensation; the needle core was then advanced while withdrawing the outer cannula, leaving the suture *in situ*. Treatments were scheduled every two weeks for a total of four sessions. Accordingly, each participant attended five study visits: visit 1 was the baseline assessment, and visits 2–5 were conducted two weeks after treatment sessions 1–4, respectively. Patients were not placed on strict diets or exercise regimens but were simply advised to maintain a healthy lifestyle.

#### 2.3.2 Control group

These participants received no active or sham intervention. They continued their usual diet and physical activity, and their weight was recorded at the same biweekly intervals as the ACE group's follow-up schedule.

### 2.4 Outcome measurement

The primary endpoint was BMI (kg/m^2^), measured at baseline (visit 1) and at four post-treatment visits (visits 2–5), each scheduled two weeks after the corresponding ACE session. Height was recorded once at enrolment, and weight was measured in the morning after an overnight fast, with light clothing and no shoes, using calibrated scales.

### 2.5 Data collection

For the ACE group, we extracted data from electronic medical records, including age, height, weight, BMI, waist circumference, and treatment dates. Only patients with complete records for all five time points were included. For participants in the control group, weight was measured prospectively at each visit. The variables routinely available and extracted were limited to age, height, weight, BMI, waist circumference, and treatment dates. Dietary caloric intake, physical activity, socioeconomic characteristics, previous weight-loss attempts, anti-obesity medication use, menopausal status, metabolic comorbidities, insulin resistance, PCOS status, and smoking were not systematically recorded in the electronic medical records or in the prospective control assessments. These variables were therefore not included in the baseline table and could not be adjusted for in the analyses.

### 2.6 Sample size and precision

This was a retrospective cohort study, and no *a priori* sample size calculation was performed. A retrospective precision analysis was therefore conducted for the primary outcome (between-group difference in BMI). With 98 participants in the ACE group and 33 participants in the congtrol group (allocation ratio approximately 3:1), a two-sided alpha of 0.05, and 80% power, a two-sample t-test approximation indicated that the study could detect a standardized effect size (Cohen's d) of approximately 0.56. Assuming a standard deviation of 3.0–4.0 kg/m^2^ for BMI, this corresponds to a detectable between-group difference of approximately 1.7–2.2 kg/m^2^. The pooled effect size reported in a recent meta-analysis of ACE for obese women was −1.72 kg/m^2^ (11), which is at the margin of this detectable range.

### 2.7 Safety observation

An adverse event was defined as any untoward medical occurrence temporally associated with ACE, whether or not considered treatment-related. Serious adverse events were defined as death, life-threatening events, hospitalization or prolongation of hospitalization, persistent or significant disability, or intervention required to prevent permanent impairment. Safety data were ascertained retrospectively from electronic medical records, including procedure notes, nursing records, and follow-up entries. For each documented event, severity (mild, moderate, or severe), duration, and outcome were recorded. The denominator was all participants in the ACE group who completed four sessions.

### 2.8 Statistical analysis

Because continuous variables deviated from normality (Shapiro–Wilk test), we summarised them as median (Q1, Q3) (15). Baseline differences were evaluated with the Mann–Whitney U test. For between-group BMI comparisons at each time point, we used a two-way ANOVA. Within-group changes were assessed using Šídák's multiple comparisons test, with the first measurement as reference (16). All tests were two-sided, and *P* < 0.05 was considered significant. Analyses were performed with GraphPad Prism 9.5.1.

## 3 Results

### 3.1 Participant characteristics

A total of 131 obese women were included: 98 in the ACE group and 33 in the control group. The flow of participants is shown in Figure 1. As summarised in Table 1, the two groups had similar baseline values for age, height, weight, BMI, and waist circumference (all *P* > 0.05). Other potential confounders were not systematically collected.

**Figure 1:**
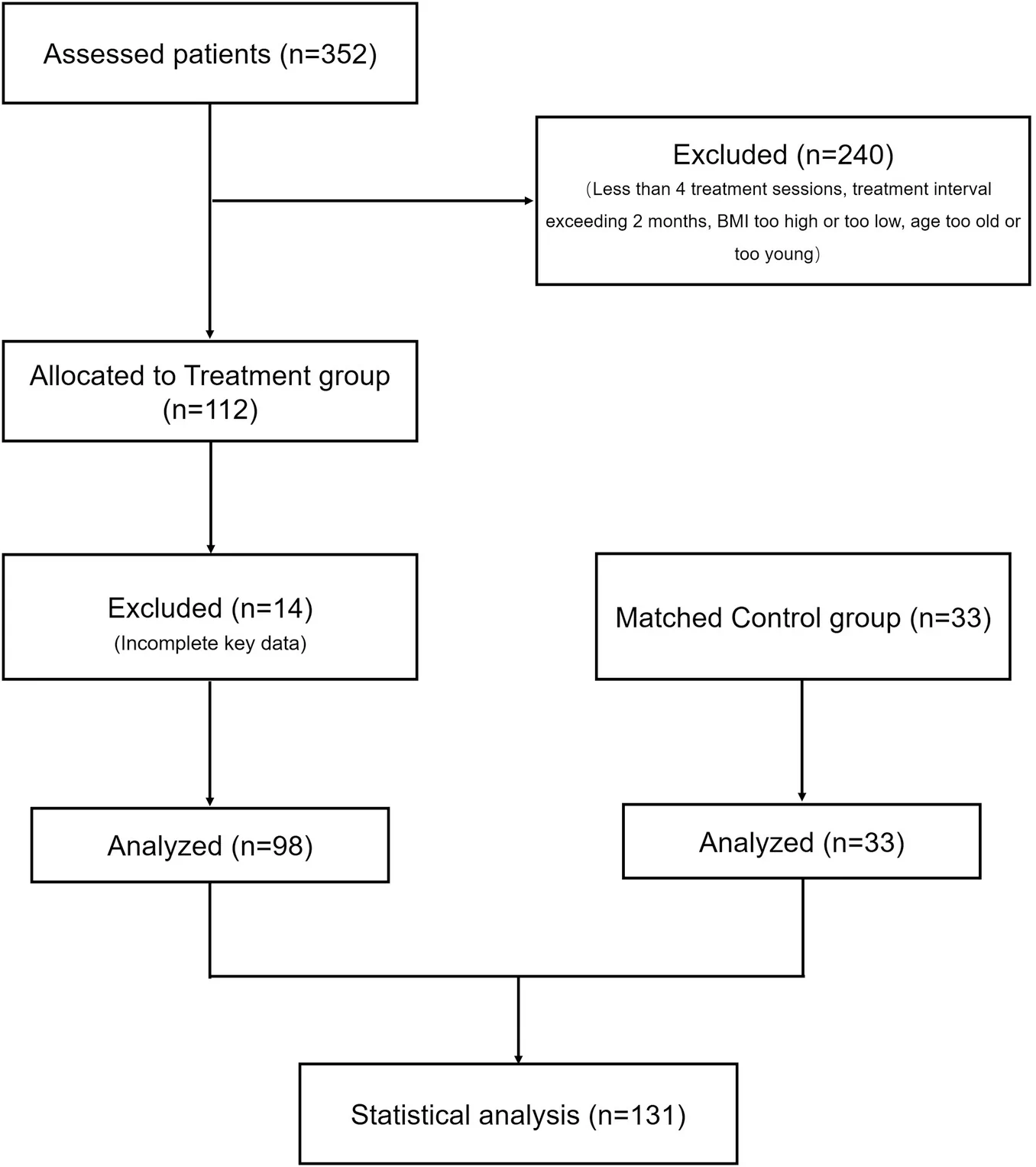
Flow diagram of participant selection and group allocation.

**Table 1:** Baseline characteristics of the study population.

Characteristic	Control group (*n* = 33)	ACE group (*n* = 98)	*P* value
Age (years)	38 (28.5, 46.5)	34 (28.8, 45)	0.297
Height (cm)	162 (160, 167)	163 (160, 167)	0.746
Body weight (kg)	81.2 (76.1, 90)	83.6 (78.1, 91.1)	0.461
BMI (kg/m^2^)	31.2 (29.4, 33.9)	31.3 (29.6, 33.9)	0.828
Waist circumference (cm)	101 (95.5, 108)	101 (97, 106)	0.692

BMI, body mass index; ACE, acupoint catgut embedding. Only variables listed above were routinely available.

### 3.2 Between-group BMI changes

Table 2 and Figure 2 display the BMI trajectories. In the control group, BMI remained virtually flat across all five visits. In contrast, the ACE group showed a steady downward trend. At baseline, the two groups were nearly identical (*P* > 0.99). After one session (visit 2), ACE-treated women had slightly lower BMI, but the difference was not yet significant (*P* = 0.488). From visit 3 onwards, the gap widened and became statistically significant (visit 3: *P* = 0.043; visit 4: *P* = 0.001; visit 5: *P* < 0.001). These results indicate that meaningful BMI reduction emerges after the second session (visit 3) and becomes more pronounced with additional treatments.

**Table 2:** Comparison of BMI between the two groups at each time point.

Time point	Control group (*n* = 33)	ACE group (*n* = 98)	*P* value
1st (baseline)	31.2 (29.4, 33.9)	31.3 (29.6, 33.9)	0.828
2nd	31.2 (29.4, 33.8)	30.7 (28.9, 33.1)	0.488
3rd	31.2 (29.4, 33.7)	29.8 (28.4, 32.5)	**0**.**043**
4th	31.2 (29.4, 34)	29.2 (27.7, 31.9)	**0**.**001**
5th	31.2 (29.4, 34.3)	29 (27.1, 31.3)	**<0**.**001**

Data are presented as median (first quartile, third quartile). ACE, acupoint catgut embedding. Visit 1 = baseline; visits 2–5 = after ACE sessions 1–4, respectively.

**Figure 2:**
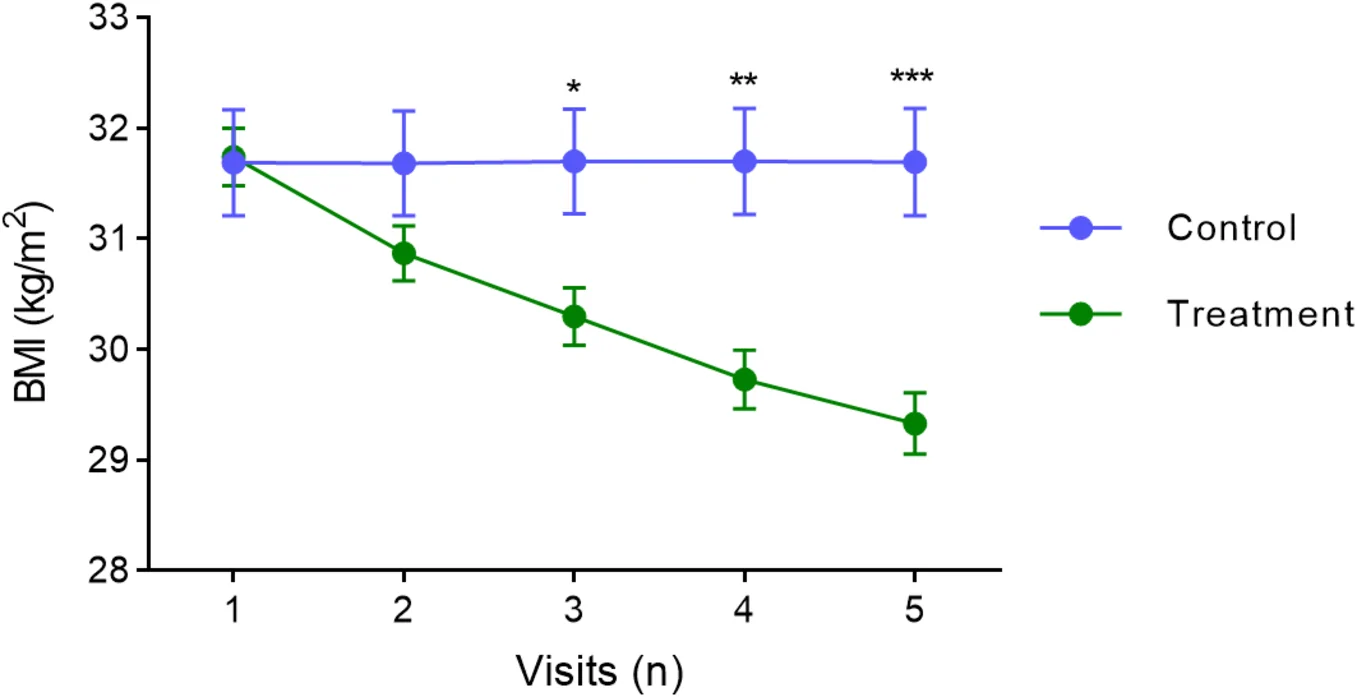
Box plots showing median BMI (with interquartile range) for the control and ACE groups at each of the five visits. *P* values indicate between-group differences (two-way ANOVA). Visit 1 = baseline; visits 2–5 = after ACE sessions 1–4, respectively.

### 3.3 Between-group changes in body weight and waist circumference

Similar patterns were observed for body weight and waist circumference (Tables 3, 4). Body weight was significantly lower in the ACE group at visits 4 and 5 (*P* = 0.049 and *P* = 0.009), whereas waist circumference differed from visit 2 onward (*P* = 0.021 at visit 2 and *P* < 0.001 thereafter). By visit 5, median reductions in the ACE group were 6.2 kg and 6.5 cm, respectively, while articipants in the control group remained stable. Between-group differences in body-weight change from baseline are presented in Supplementary Table S1. The difference (ACE minus control) increased progressively from −1.88 kg at visit 2 to −3.53 kg at visit 3, −4.6 kg at visit 4, and −5.55 kg at visit 5.

**Table 3:** Comparison of body weight between the two groups at each time point.

Time point	Control group (*n* = 33)	ACE group (*n* = 98)	*P* value
1st (baseline)	81.2 (76.1, 90)	83.6 (78.1, 91.1)	0.461
2nd	81.3 (76.1, 89.8)	81.3 (76.7, 87.7)	0.87
3rd	81.7 (76.1, 90.3)	79.9 (74, 85.9)	0.315
4th	81.3 (76.1, 89.4)	78.5 (72.8, 84.6)	**0**.**049**
5th	81.2 (76.1, 89.4)	77.4 (71.5, 83.6)	**0**.**009**

Data are presented as median (first quartile, third quartile). ACE, acupoint catgut embedding. Visit 1 = baseline; visits 2–5 = after ACE sessions 1–4, respectively.

**Table 4:** Comparison of waist circumference between the two groups at each time point.

Time point	Control group (*n* = 33)	ACE group (*n* = 98)	*P* value
1st (baseline)	101 (95.5, 108)	101 (97, 106)	0.692
2nd	103 (95.5, 107)	97.5 (94, 102)	**0**.**021**
3rd	101 (95.9, 107)	96 (91, 101)	**<0**.**001**
4th	101 (95.5, 107)	95 (90, 99.1)	**<0**.**001**
5th	102 (95.5, 107)	94.5 (89, 99)	**<0**.**001**

Data are presented as median (first quartile, third quartile). ACE, acupoint catgut embedding. Visit 1 = baseline; visits 2–5 = after ACE sessions 1–4, respectively.

### 3.4 Within-group changes

Figure 3 plots the change from baseline for each group. For the control group, none of the subsequent visits differed significantly from baseline. For the ACE group, however, every post-treatment measurement (visits 2 through 5) was significantly lower than baseline (all *P* < 0.05). This confirms that the weight loss is not spontaneous but treatment-driven, and that the benefit increases progressively with successive ACE sessions.

**Figure 3:**
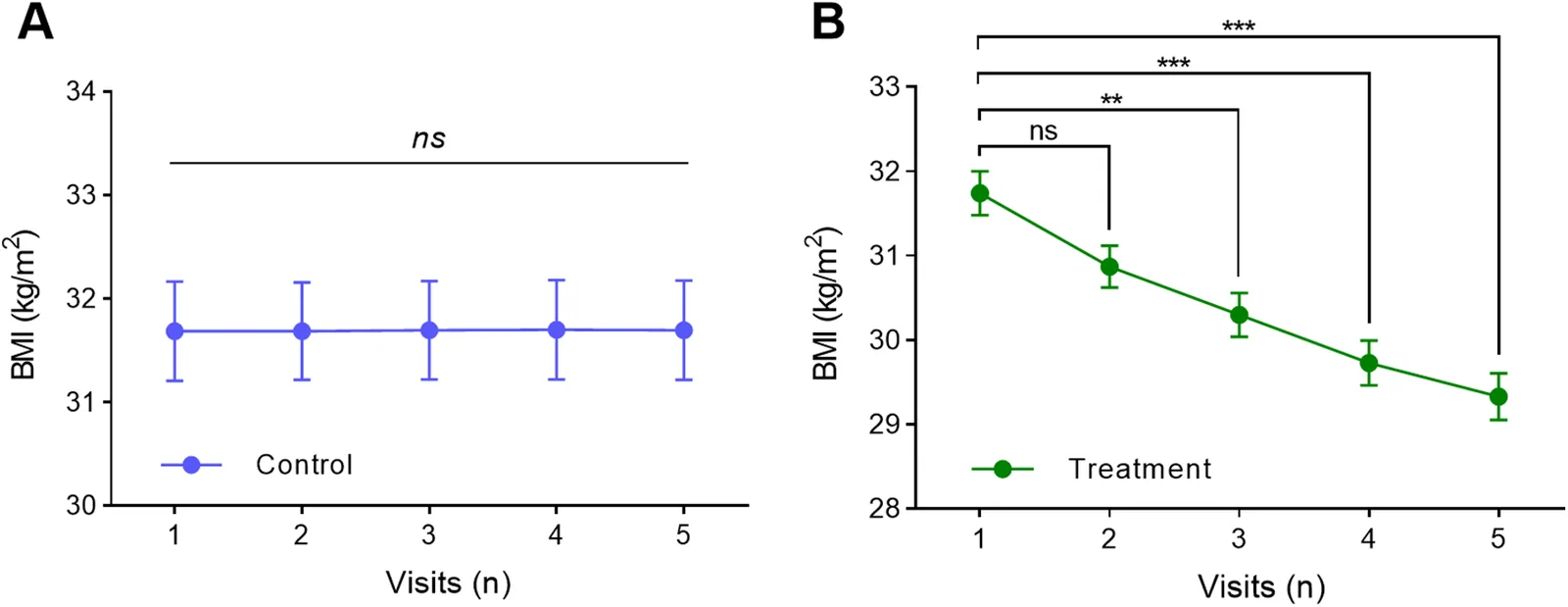
Within-group changes in BMI from baseline. Panel **A**: control group (all comparisons non-significant). Panel **B**: ACE group (all post-baseline visits showed significant reductions, *P* < 0.05). ns = not significant. Visit 1 = baseline; visits 2–5 = after ACE sessions 1–4, respectively.

### 3.5 Safety

An adverse event was defined as any untoward medical occurrence temporally associated with ACE, whether or not considered treatment-related. No major adverse events were identified during the observed treatment period (0/98). Minor local reactions—soreness, distension, or bruising at the embedding sites—were documented in a few patients; these were mild, lasted a few days, and resolved spontaneously. No allergic or systemic adverse events were documented.

## 4 Discussion

Three main findings emerged from this real-world cohort study. First, ACE treatment may have reduced BMI in obese women compared with no treatment. Second, the reduction became statistically appreciable only after the third visit (i.e., after two treatment sessions) and grew larger with each additional session. Third, untreated participants showed no meaningful weight change over the same observation period, ruling out secular trends. Collectively, these observations provide evidence consistent with a cumulative weight-lowering effect of ACE in this population, although residual confounding cannot be excluded. The magnitude of the effect—a median BMI drop of 2.3 kg/m^2^ over approximately two months—is clinically relevant. It aligns well with the pooled estimate from a recent meta-analysis and even exceeds it slightly (11). This might be because our treatment course was longer (four sessions vs. typically three in many trials) and because we used an untreated control rather than a sham, which can eliminate any “placebo” subtraction and better reflect the net benefit in everyday practice. Moreover, our participants were not placed on restrictive diets, so the observed loss is likely attributable to the ACE itself rather than to concomitant lifestyle changes.

Consistent with the BMI findings, body weight and waist circumference also improved in the ACE group. The earlier response of waist circumference than BMI and body weight suggests that ACE may preferentially affect abdominal obesity. This could be because waist circumference largely reflects central and visceral fat and abdominal fluid retention, which may respond more rapidly to ACE-induced modulation of gastrointestinal motility, autonomic tone, and local adipose metabolism.

The cumulative nature of the effect is particularly noteworthy. The fact that one session produced only a non-significant trend, whereas two, three, and four sessions yielded clear and growing differences, is consistent with a cumulative association with successive sessions; however, because all participants received the same schedule, this cannot be interpreted as a dose-response relationship. This carries practical implications: clinicians should counsel patients that results may not be immediate and that a minimum of two to four sessions is needed to achieve meaningful change. This pattern is consistent with other studies that examined different treatment frequencies, which also pointed to a threshold effect (17). It also aligns with the traditional view of acupuncture as a therapy that requires repeated stimulation to accumulate therapeutic impact.

How might ACE produce these effects? The sustained mechanical stimulus on points like Zusanli, Zhongwan, and Tianshu is believed to regulate the spleen-stomach axis, transform dampness, and move *qi*—all concepts rooted in traditional Chinese medicine (13, 18, 19). At the molecular level, ACE has been shown to dampen hypothalamic appetite signals, reduce ghrelin, enhance leptin sensitivity, and increase energy expenditure via brown adipose tissue activation (20, 21). It also improves insulin resistance and lipid profiles, although the effects on HDL and LDL are less consistent (11, 22). In addition, ACE may influence female reproductive hormones, which could be particularly relevant for obese women with polycystic ovary syndrome (23, 24).

From a clinical standpoint, ACE offers several advantages. It is minimally invasive, low-cost relative to long-term pharmacotherapy, and does not require the stringent adherence demanded by diet-and-exercise regimens. For patients who have failed or cannot tolerate GLP-1 agonists, or who are not candidates for surgery, ACE could fill an important therapeutic niche. Nonetheless, we stress that ACE should be viewed as part of a comprehensive approach; combining it with healthy dietary and physical activity habits would likely optimise and prolong the benefits (25, 26).

We acknowledge several limitations. The retrospective design and non-randomised allocation introduce the possibility of confounding, although baseline age and BMI were similar between groups. Several factors that may affect weight trajectory were not systematically collected, including dietary caloric intake, physical activity, socioeconomic characteristics, previous weight-loss attempts, anti-obesity medication use, menopausal status, metabolic comorbidities, insulin resistance, PCOS status, and smoking. Although participants were not placed on strict diets or exercise regimens and participants in the control group had no concurrent weight-loss interventions, differences in these unmeasured factors could substantially confound the observed BMI changes. Residual confounding therefore cannot be excluded, and the findings should be interpreted as associational rather than causal. The control group was smaller than the ACE group, which might affect the precision of some comparisons. No *a priori* sample size calculation was performed, and the final sample comprised 98 participants in the ACE group and 33 participants in the control group (approximately 3:1, imbalanced). Precision for the primary BMI comparison was limited, subgroup and safety analyses were underpowered, rare adverse events could not be excluded. Furthermore, the ACE group was retrospectively recruited from 2016 to 2025, whereas the control group was prospectively enrolled from 2025 to 2026; this temporal disparity could introduce unmeasured period effects, such as secular changes in lifestyle behaviours or the lingering impact of the COVID-19 pandemic, which might have influenced weight changes independently of the intervention. We did not include a sham-ACE arm, so we cannot completely exclude a placebo contribution. Our sample comprised only women from a single tertiary centre, limiting generalisability to men or other ethnic groups. Finally, follow-up ended at visit 5, so we do not know whether the weight loss was sustained after treatment stopped.

## 5 Conclusions

This retrospective cohort study provides some evidence suggesting that ACE may be associated with weight loss in obese women. The association appeared to become detectable after two sessions and to strengthen with additional treatments. Given that no major adverse events were identified during the observed treatment period in this cohort, ACE may be considered as a possible complementary option for obesity management, particularly for patients who cannot access or do not respond to existing pharmacological or surgical therapies. However, the sample size was limited, the allocation ratio was imbalanced, and the study was underpowered for subgroup and safety analyses. These findings should therefore be considered preliminary and hypothesis-generating. Randomised controlled trials with extended follow-up and different treatment schedules are needed to confirm these findings and to refine treatment protocols.

## Data Availability

The original contributions presented in the study are included in the article/Supplementary Material, further inquiries can be directed to the corresponding authors.

## References

[1] Pan XF Fang ZZ Zhang L Pan A. Obesity in China: current progress and future prospects. Lancet Diabetes Endocrinol. (2026) 14:178–86. 10.1016/S2213-8587(25)00357-2 41389801

[2] Song C Shen H. Obesity control and cancer prevention in China: insights from the weight management year. China CDC Wkly. (2025) 7:1127–31. 10.46234/ccdcw2025.189 40937167 PMC12421416

[3] Nowak K Paluch M Cudzik M Syska K Gawlikowska W Janczura J. From steatosis to cirrhosis: the role of obesity in the progression of liver disease. J Diabetes Metab Disord. (2025) 24:227. 10.1007/s40200-025-01754-x 41104308 PMC12521716

[4] Jatin CP Jatin CP Beniwal SS Pithalla LB Dwivedi A Shetty VR. Managing obstetric and gynecologic complications in obese women: evidence-based strategies for optimizing outcomes and reducing risks. Ann Med Surg (Lond). (2025) 87:3586–95. 10.1097/MS9.0000000000003300 40486547 PMC12140740

[5] Ahmed IS Tapponi SL Manaa HM Soltani A Hussein ES Parvaresh FA. A narrative review of weight management strategies: from lifestyle interventions to emerging pharmacotherapies. J Obes. (2026) 2026:e6556057. 10.1155/jobe/6556057 41973072 PMC13075284

[6] Kolli RT Aoutla S Jyothi N Mohamed Kalifa MRH Raju A Cheenikkal Muralidharan K. Rebound or retention: a meta-analysis of weight regain after the discontinuation of glucagon-like peptide-1 (GLP-1) receptor agonists and other anti-obesity drugs. Cureus. (2025) 17:e94926. 10.7759/cureus.94926 41116804 PMC12535773

[7] Lee I Kim J Alkhaldi A Alayed SSA Fallata EM Eledreesi M. Metabolic and bariatric surgery for class I obesity [body mass index 30-34.9kg/m(2)]: a comprehensive literature review. J Metab Bariatr Surg. (2025) 14:165–73. 10.17476/jmbs.2025.14.3.165 41568127 PMC12817832

[8] Zhang X Long D Chen J Lei Z Hu J Chen Y. Effectiveness and safety of acupoint catgut embedding for patients with mild psoriasis and overweight: study protocol of a multicenter double-blind randomized controlled trial. Front Med (Lausanne). (2026) 13:1796602. 10.3389/fmed.2026.1796602 41939750 PMC13043425

[9] Li Y Zhou F Zhang H Chen X Yin M Tang M. Acupoint catgut embedding therapy in traditional Chinese medicine for managing allergic rhinitis. J Vis Exp. (2024):e67037. 10.3791/67037 39760374

[10] Yan R Liu X Bai J Yu J Gu J. Influence of catgut implantation at acupoints on leptin and insulin resistance in simple obesity rats. J Tradit Chin Med. (2012) 32:477–81. 10.1016/s0254-6272(13)60058-8 23297576

[11] Li S Zhou H Zhou M Lou L Luo L Ji S. The effects of acupoint catgut embedding therapy on anthropometric parameters and endocrine function in obese women: a systematic review and meta-analysis. Front Nutr. (2025) 12:1583556. 10.3389/fnut.2025.1583556 40667439 PMC12259452

[12] Jin Y Huang Y Zhu J Liao D Zeng S Jin X. Acupoint catgut embedding regulates community structure of intestinal flora in central obesity during perimenopause. Women Health. (2024) 64:857–69. 10.1080/03630242.2024.2422876 39496462

[13] Kazemi AH Adel-Mehraban MS Jamali Dastjerdi M Alipour R. A comprehensive practical review of acupoint embedding as a semi-permanent acupuncture: a mini review. Medicine (Baltimore). (2024) 103:e38314. 10.1097/MD.0000000000038314 38847703 PMC11155527

[14] Fan H Kouvari M Guo C Liu Z Zhang X Wang H. A comprehensive comparison of two commonly used BMI thresholds for non-communicable diseases and multimorbidity in the Chinese population. Clin Nutr. (2025) 48:70–9. 10.1016/j.clnu.2025.03.016 40154198

[15] Green DJ Campbell MJ Koutoumanou E. When means and standard deviations are an incomplete summary of a continuous variable: problems, solutions, and utilising the reference ranges to check normality. BMJ Med. (2026) 5:e001796. 10.1136/bmjmed-2025-001796 41658050 PMC12878307

[16] Tai C Ling C Yang Y Zhang B Sun J Mo N. Tie2-expressing monocytes/macrophages promote angiogenesis in chronically ischaemic brain tissue. Cell Biosci. (2025) 15:62. 10.1186/s13578-025-01401-1 40399921 PMC12093791

[17] Chen X Huang W Wei D Zhao J-P Zhang W Ding D-G. Effect of acupoint catgut embedding for middle-aged obesity: a multicentre, randomised, sham-controlled trial. Evid Based Complement Alternat Med. (2022) 2022:1–19. 10.1155/2022/4780019 PMC8898815 35265146

[18] Chen X Huang W Hu F Jin YT Hong ZH Zhou ZY. Regularity of acupoint selection for simple obesity treated by acupoint catgut embedding based on complex network technology. Zhen Ci Yan Jiu. (2018) 43:585–90. 10.13702/j.1000-0607.170448 30232869

[19] Huiyan Z Purumea J Chaewon L Chang-Hyun H. Acupoint catgut embedding for simple obesity in animal studies: a systematic review and meta-analysis. J Tradit Chin Med. (2023) 43:860–67. 10.19852/j.cnki.jtcm.2023.05.002 37679973 PMC10465837

[20] Inprasit C Huang YC Lin YW. Evidence for acupoint catgut embedding treatment and TRPV1 gene deletion increasing weight control in murine model. Int J Mol Med. (2020) 45:779–92. 10.3892/ijmm.2020.4462 31922226 PMC7015137

[21] Xiong Y Wang X Gong M Ji Q Li Y Hu A. Acupoints catgut embedding recovers leptin resistance via improving autophagy progress mediated by AMPK-mTOR signaling in obese mice. Heliyon. (2024) 10:e29094. 10.1016/j.heliyon.2024.e29094 38623207 PMC11016596

[22] Yin N Wu X Ren R Luo J. The effect of acupoint catgut embedding and drug therapy on hyperlipidemia: a meta-analysis of randomized controlled trials. Biotechnol Genet Eng Rev. (2024) 40:664–77. 10.1080/02648725.2023.2186559 36880486

[23] Li P Lu Y Wang Y Weng A Liang X. Effects of different acupuncture methods on polycystic ovarian syndrome: a systematic review and network meta-analysis. BMC Complement Med Ther. (2026) 26:104. 10.1186/s12906-026-05295-5 41680702 PMC13005429

[24] Liang Y Yuan Y Yang J. Acupoint catgut embedding: a potential intervention strategy for obesity-related precocious puberty. Front Endocrinol (Lausanne). (2024) 15:1448111. 10.3389/fendo.2024.1448111 39469573 PMC11513323

[25] Yue J- Li X- Zhang Y- Yang G- Mah JZ- Li A. Comparing verum and sham acupoint catgut embedding for adults with obesity: a systematic review and meta-analysis of randomized clinical trials. Medicine (Baltimore). (2024) 103:e36653. 10.1097/MD.0000000000036653 38277544 PMC10817137

[26] Huang LC Pan WY. Comparation of effect and cost-benefit analysis between acupoint catgut-embedding and electroacupuncture on simple obesity. Zhongguo Zhen Jiu. (2011) 31:883–6. 10.1097/MOP.0b013e328341d1da 22043672

