# Supplementary material for: Cumulative weight reduction across repeated acupoint catgut embedding sessions in obese women: a retrospective cohort study
Source: Front Med (Lausanne). 2026 Oct 16;13:1945172. doi: 10.3389/fmed.2026.1945172 (PMC13719440; doi:10.3389/fmed.2026.1945172)
Supplement: Supplementary file 1 [file Table1.pdf]

Supplementary Materials

Table S1. Comparison of body weight differences between the two groups after four treatment sessions.

**Visit**	**Control group (n = 33)**	**ACE group (n = 98)**	***P* value**
2nd	0 (-0.23, 0.2)	-1.88 (-3.45, -0.9)	**<0.001**
3rd	0 (0, 0.1)	-3.53 (-5.05, -2.31)	**<0.001**
4th	0 (0, 0.1)	-4.6 (-7.46, -2.94)	**<0.001**
5th	0 (0, 0.2)	-5.55 (-8.65, -3.48)	**<0.001**

Data are presented as median (first quartile, third quartile). ACE, acupoint catgut embedding. Visit 1 = baseline; visits 2–5 = after ACE sessions 1–4, respectively.
